# A Thermotolerant Marine *Bacillus amyloliquefaciens* S185 Producing Iturin A5 for Antifungal Activity against *Fusarium oxysporum* f. sp. *cubense*

**DOI:** 10.3390/md19090516

**Published:** 2021-09-11

**Authors:** Pratiksha Singh, Jin Xie, Yanhua Qi, Qijian Qin, Cheng Jin, Bin Wang, Wenxia Fang

**Affiliations:** 1 State Key Laboratory of Non-Food Biomass and Enzyme Technology, Guangxi Academy of Sciences, Nanning 530007, China; pratiksha23@gxas.cn (P.S.); xiejin@gxas.cn (J.X.); qiyanhua@gxas.cn (Y.Q.); qqij@gxas.cn (Q.Q.); jinc@im.ac.cn (C.J.); 2 National Engineering Research Center for Non-Food Biorefinery, Guangxi Academy of Sciences, Nanning 530007, China; 3 State Key Laboratory of Mycology, Institute of Microbiology, Chinese Academy of Sciences, Beijing 100101, China

**Keywords:** antifungal activity, *Bacillus amyloliquefaciens*, Panama disease, *Fusarium oxysporum* f. sp. *cubense*, bioactive compound, iturin A5

## Abstract

Fusarium wilt of banana (also known as Panama disease), is a severe fungal disease caused by soil-borne *Fusarium oxysporum* f. sp. *cubense* (*Foc*). In recent years, biocontrol strategies using antifungal microorganisms from various niches and their related bioactive compounds have been used to prevent and control Panama disease. Here, a thermotolerant marine strain S185 was identified as *Bacillus amyloliquefaciens*, displaying strong antifungal activity against *Foc*. The strain S185 possesses multiple plant growth-promoting (PGP) and biocontrol utility properties, such as producing indole acetic acid (IAA) and ammonia, assimilating various carbon sources, tolerating pH of 4 to 9, temperature of 20 to 50 °C, and salt stress of 1 to 5%. Inoculation of S185 colonized the banana plants effectively and was mainly located in leaf and root tissues. To further investigate the antifungal components, compounds were extracted, fractionated, and purified. One compound, inhibiting *Foc* with minimum inhibitory concentrations (MICs) of 25 μg/disk, was identified as iturin A5 by high-resolution electrospray ionization mass spectrometry (HR-ESI-MS) and nuclear magnetic resonance (NMR). The isolated iturin, A5, resulted in severe morphological changes during spore germination and hyphae growth of *Foc*. These results specify that *B. amyloliquefaciens* S185 plays a key role in preventing the *Foc* pathogen by producing the antifungal compound iturin A5, and possesses potential as a cost-effective and sustainable biocontrol strain for Panama disease in the future. This is the first report of isolation of the antifungal compound iturin A5 from thermotolerant marine *B. amyloliquefaciens* S185.

## 1. Introduction

Crop wastage is primarily affected by plant diseases, which are mostly caused by soil-borne fungi leading to substantial yield reduction and income losses [1]. Banana (*Musa* spp.) is a major cash crop in the tropics and subtropics and is one of the top ten staple foods in the world [2,3]. The majority of vegetatively propagated bananas are susceptible to many pests and diseases, notably Fusarium wilt of banana (FWB). This disease is caused by the soil-borne fungus *Fusarium oxysporum* f. sp. *cubense* (*Foc*) and is recognized as the most damaging and extensively disseminated disease in banana-producing regions worldwide [4,5]. FWB has spread to the world’s leading banana-producing areas since the 1990s [6]. In China, FWB has expanded over much in recent years, placing the banana industry’s health and long-term sustainability under risk [7]. As the pathogen of FWB, *Foc* is resistant to stress and capable of living in soil for up to 30 years [8]. *Foc* infects the xylem through the roots, resulting in widespread necrosis, plant mortality, and causing widespread orchard damage [4,6].

Currently, no suitable cultivar has been selected or bred to reduce the occurrence of FWB [9]. This is a worldwide issue with consequences that include losses in a wide variety of banana production methods and plant mortality in certain cases. Crop rotation, the selection of resistant varieties, and chemical or biological approaches are now the most common strategies for managing FWB [10,11]. However, due to the persistent spread of FWB throughout continents, countries, and regions, there is no effective control system [12]. Chemical fungicides are (relatively) the most effective tool for controlling this soil-borne disease [13]. However, scientists have currently found that long-term usage of fungicides might result in negative consequences such as environmental pollution, plant disease outbreaks, escalating production costs due to chemical overuse, and even human toxicity [14]. So far, there has not been a feasible way to manage the *Foc* pathogen in the field. Soil sterilization with fungicides has only been utilized on a small scale in intensive agriculture such as in greenhouses [6,15]. Therefore, ecologically and environmentally friendly cultural practices are urgently required [16]. In present agriculture, the biocontrol approach has been being investigated for several years, and many helpful bacteria have been proposed for controlling crop diseases. In addition, biocontrol is the only alternative disease-management strategy that explores plant growth-promoting (PGP) antagonistic bacteria or fungi that are regarded to be safe, ecologically friendly, and cost-effective [11,17], whereas many biocontrol agents are selective to host species, pathogen type, environmental circumstances, soil types, seasons, etc. [18]. Hence, a comprehensive study is necessary to find novel biocontrol agents that can respond to a wide range of environments [19].

The marine ecosystem is composed of marine invertebrates, plants, and their related microbial communities, providing a potential source for new bioactive compounds [20]. The marine microorganisms in the marine ecosystem produce the majority of secondary metabolites which have a wide range of functions, including antiviral, antifungal, immune-suppressive, anti-inflammatory, antitumor, and many other biotechnological and pharmaceutical applications [21,22]. The trend of discovering novel compounds from marine microbes has been increasing during the past decade [23]. Several strains of *Bacillus* spp., which have a suppressive effect against plant diseases caused by soil-borne phytopathogens, have expanded rapidly, and interested readers now have vast quantities of information on biocontrol strategies, as well as their applications and efficacies under a wide range of situations [24]. *Bacillus* spp. are chosen in agricultural systems due to their capacity to produce endospores that can withstand heat and desiccation, in addition to their capacity to be formed into stable dry powders with long shelf lives [25]. Furthermore, many *Bacillus* species have fast growth rates, common inhabitation of plant root microflora, and the ability to produce a large number of secondary metabolites, which are important in antibiosis against a variety of harmful pathogens [26]. Biocontrol methods using *Bacillus* spp. are divided into two categories: direct antagonism and indirect antagonism. Nutrition and geographical location competition, production of secondary metabolites with antibiosis activity, secretion of hydrolases, and the emission of volatile organic molecules are examples of direct antagonism, whereas indirect antagonism involves changing the variety of the soil or plant microbial population to induce plant tolerance and prevent pathogens [10]. *Bacillus* produces important antagonistic substances, such as surfactin [27], iturins [28], and fengycin [29], which are usually generated by non-ribosomal peptide synthetase (NRPS) and inducing plant eliciting system against infections [30].

In this study, we isolated a *B. amyloliquefaciens* strain S185 from offshore of the South Sea, China. This strain exhibits antagonism activity against *Foc*, possesses PGP traits and has other properties suitable for biocontrol utilization. Further analysis showed that iturin A5 is the main bioactive compound contributing to antifungal activity. To the best of our knowledge, this is the first report on the inhibition of *Foc* growth by compound iturin A5 extracted from a marine *B. amyloliquefaciens* strain, which is a potential biocontrol strain for FWB.

## 2. Results

### 2.1. A Thermotolerant Marine Bacterium S185 Possesses Antagonistic Activity against Foc

**Figure 1:**
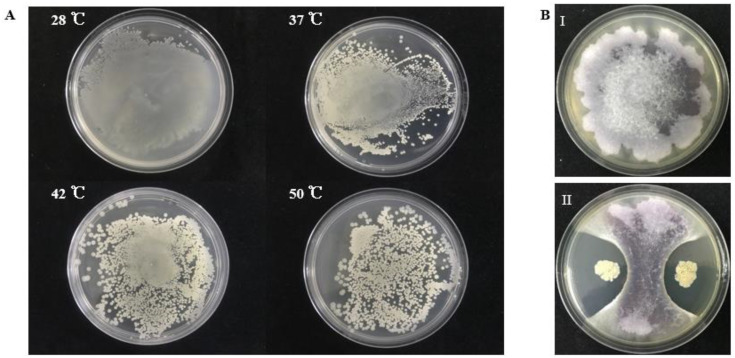
Thermotolerant property of S185 and its antagonistic activity towards *Foc*. (**A**). Strain S185 was cultured at 28 °C, 37 °C, 42 °C, and 50 °C, respectively. After 24 h cultivation plates were taken out for photographing. (**B**). Dual culture plate assay for the screening of strain S185 against *Foc*, (**I**). *Fusarium oxysporum* f. sp. *cubense* (*Foc*) control plate, (**II**). Growth inhibition of *Foc* pathogen by S185.

From sediment samples collected from offshore of South Sea, China, we isolated a thermotolerant bacterium S185. This strain is capable of growing at 28 °C, 37 °C, 42 °C, and 50 °C, respectively (Figure 1A). We then examined the antagonistic activity of S185 against *Foc*, the phytopathogen of FWB. As shown in Figure 1B, strain S185 displayed strong antifungal activity against *Foc* with an inhibition percentage of 78% on a dual culture plate (Table S1).

**Figure 2:**
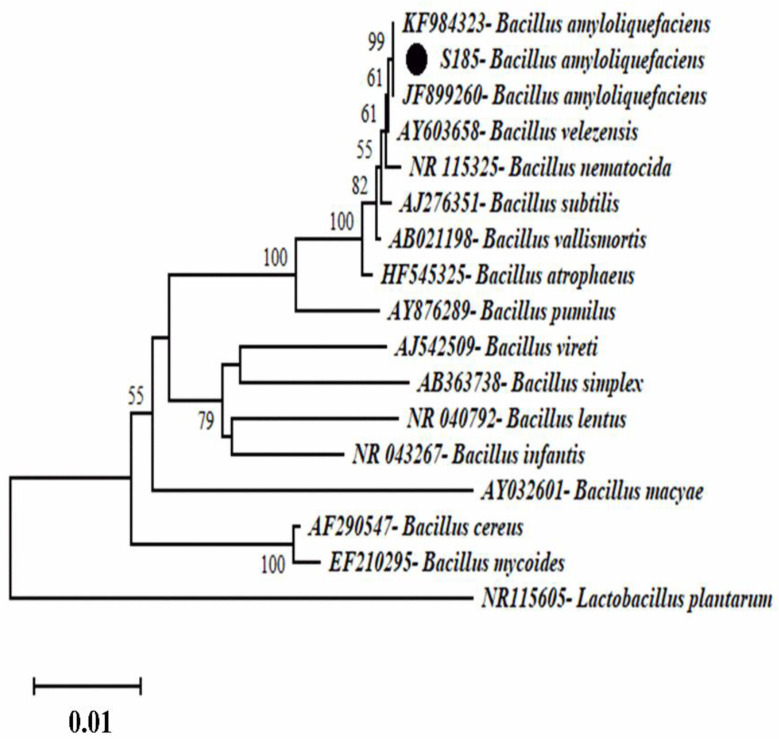
Phylogenetic tree representing the position of strain S185 compared to other strains inside the genus *Bacillus* with *Lactobacillus plantarum* as an outgroup. The tree was created via neighbor-joining with a scale bar of 0.01 substitutions per nucleotide.

Phylogenetic analysis was conducted to identify the strain S185. After PCR of the 16S *rRNA* product and sequencing analysis, the BlastN program was used to compare the 16S *rRNA* sequence of strain S185 to nucleotide sequences in the NCBI GenBank database. The strain S185 displayed over 97% similarity with the aligned species. A phylogenetic tree was constructed using representative strains from BlastN (Figure 2). The phylogenetic tree indicated that the strain was *Bacillus amyloliquefaciens*.

### 2.2. S185 Displays PGP Traits and Tolerance to Abiotic Stresses

Bacterial strains releasing secondary metabolites such as ammonia are helpful in the prevention of fungal infections in plants [31]. The S185 strain indeed showed strong ammonia production in the medium, but was negative for phosphorus (P) solubilization, hydrogen cyanide (HCN), and siderophore production (Table S1). Indole acetic acid (IAA) production is a key property of plant growth-promoting bacteria (PGPB). Strain S185 produces 93.96 ± 2.28 and 57.13 ± 1.32 µg·ml^−1^ IAA in the presence and absence of tryptophan, respectively (Table S1).

**Figure 3:**
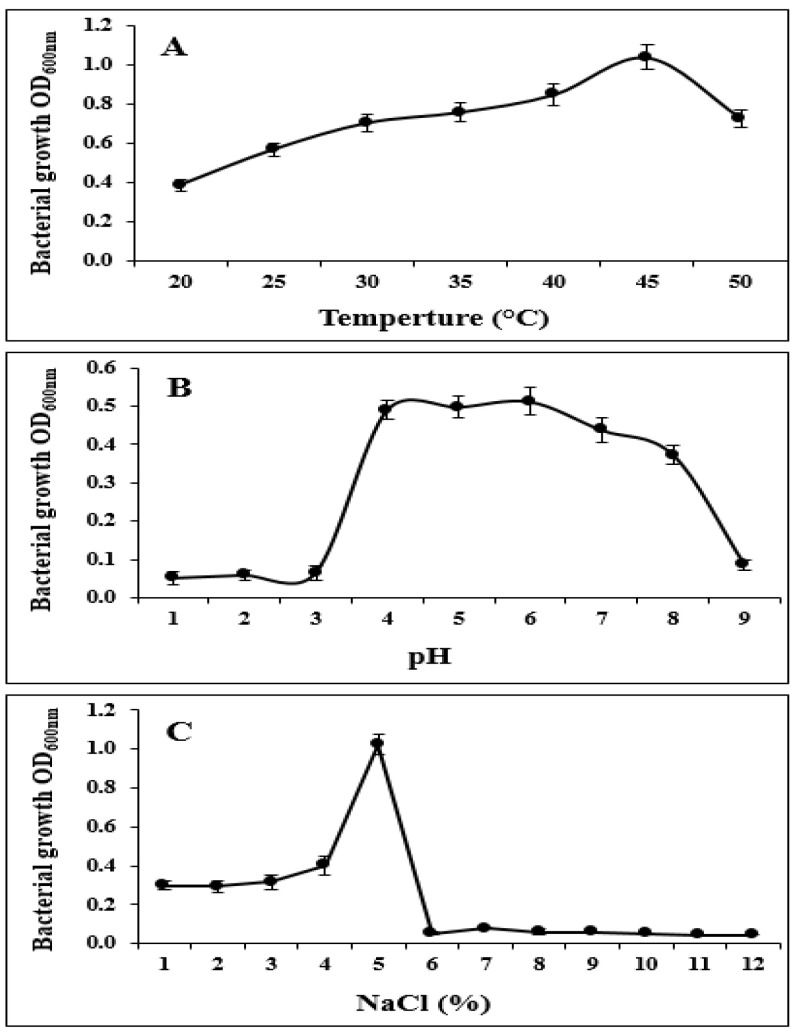
Tolerance of strain *B. amyloliquefaciens* S185 towards abiotic stresses. (**A**). Temperature (20–50 °C), (**B**). pH (1–9), (**C**). Salt (1–12%).

In addition, strain S185 exhibited tolerance to abiotic stresses such as pH of 4–9, temperature of 20–50 °C, and salt concentration of 1–5%. The optimum growth of S185 was observed at pH 6, temperature 45 °C, and 5% of NaCl (Figure 3). These results confirm that S185 displays PGP traits and tolerance to abiotic stresses.

### 2.3. S185 Utilizes Broad Carbon Substrates

Strains having a broad metabolite tolerance are more appropriate for plant nodulation [32]. Carbon substrates utilization pattern of strain S185 was tested against GNIII Biolog plate. Strain S185 was positive in the assimilation of dextrin, d-turanose, d-salicin, *N*-acetyl-d-glucosamine, *N*-acetyl-β-d-mannosamine, *N*-acetyl-d-galactosamine, *N*-acetyl neuraminic acid, d-fructose, d-galactose, 3-methyl glucose, d-fucose, l-fucose, inosine, d-sorbitol, d-mannitol, d-arabitol, myo-inositol, glycerol, d-glucose-6-PO4, d-fructose-6-PO4, d-aspartic acid, d-serine, glycyl-l-proline, l-alanine, l-arginine, l-aspartic acid, l-glutamic acid, L-histidine, L-serine, lincomycin, niaproof 4, pectin, d-gluconic acid, d-glucuronic acid, glucuronamide, mucic acid, quinic acid, tetrazolium violet, tetrazolium blue, and, l-lactic acid on Biolog GN III plate (Table S2). These findings indicate that the S185 strain is capable of using a wide range of carbon substrates.

### 2.4. S185 Colonizes in All Tissues of Banana Plants

**Figure 4:**
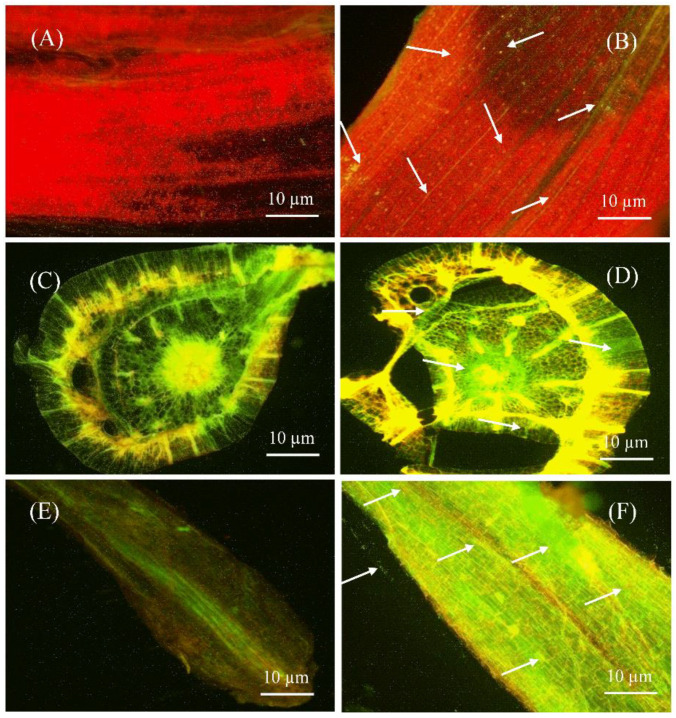
Confocal laser scanning microscopy (CLSM) showing colonization of *B. amyloliquefaciens* S185 in tissues of banana plants. (**A**,**C**,**E**) are sectioned leaf, stem, and root tissues of banana plants without S185 inoculation, (**B**,**D**,**F**) are sectioned leaf, stem, and root tissues of banana plants after 96 h of inoculation with S185. White arrow marks indicate colonization of strain S185 as small green dots in banana plants.

The capacity of bacteria to colonize plant tissues is important for disease control and plant growth development. Therefore, colonization of green fluorescent protein (GFP)-tagged S185 was assessed in tissues of banana plants by confocal laser scanning microscopy (CLSM). In control plants, without GFP-tagged S185 there was no GFP signal in leaf, stem, and root tissues of plants (Figure 4A,C,E), whereas the GFP-tagged S185 cells were observed as green spots in all plant tissues after 96 h of inoculation (Figure 4B,D,F). The strain S185 exhibited maximum colonization in roots followed by leaves and stems (Figure 4).

### 2.5. Antifungal Activity of Main Active Compound ***1*** Extracted from S185

**Figure 5:**
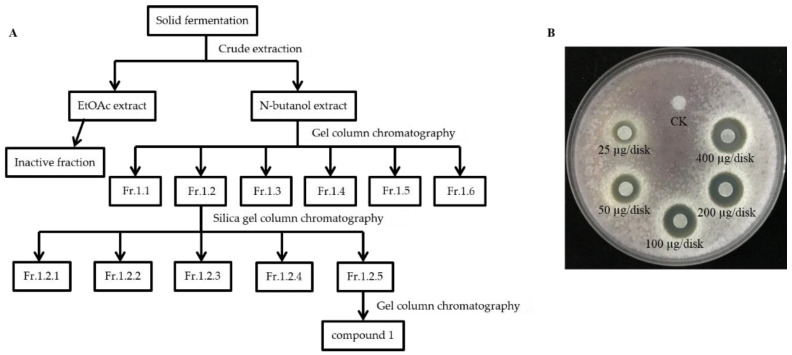
Isolation and antifungal activity of main active compound **1** of strain S185. (**A**) Flow chart showing extraction and separation procedure of compound **1** from strain S185 (**B**). Inhibitory effect of compound **1** on the growth of *Foc* at a different level of concentrations. CK; methanol control.

Despite the strong antagonistic activity of S185 against *Foc*, we next conducted crude extraction of strain S185 to identify the active components. Guided by activity tracing, butanol extracted components were further fractionated by silica gel column chromatography and gel column chromatography; finally, the main active antifungal compound **1** was isolated from S185 with the yield of 20 mg/L (Figure 5A). Compound **1** displayed the best antifungal activity as shown by the inhibition zone (Figure 5B). At the concentration of 25 μg/disk, compound **1** started to inhibit the growth of *Foc* pathogen with a clear inhibition zone of 7.67 mm (Figure 5B). The diameter of the inhibition zone increased to 9.67 mm, 12.33 mm, 13.33 mm, and 14.33 mm at the 50, 100, 200, and 400 ug/disk of compound **1**, respectively (Table S3; Figure 5B). These results confirm that compound **1** produced by strain S185 has strong antifungal activity against *Foc*.

### 2.6. Identification of Compound ***1*** as Iturin A5

**Figure 6:**
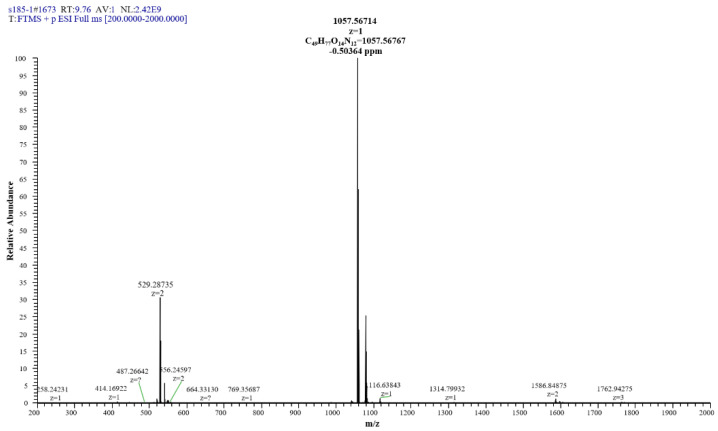
HR-ESI-MS of antifungal compound **1** isolated from *B. amyloliquefaciens* S185.

**Figure 7:**
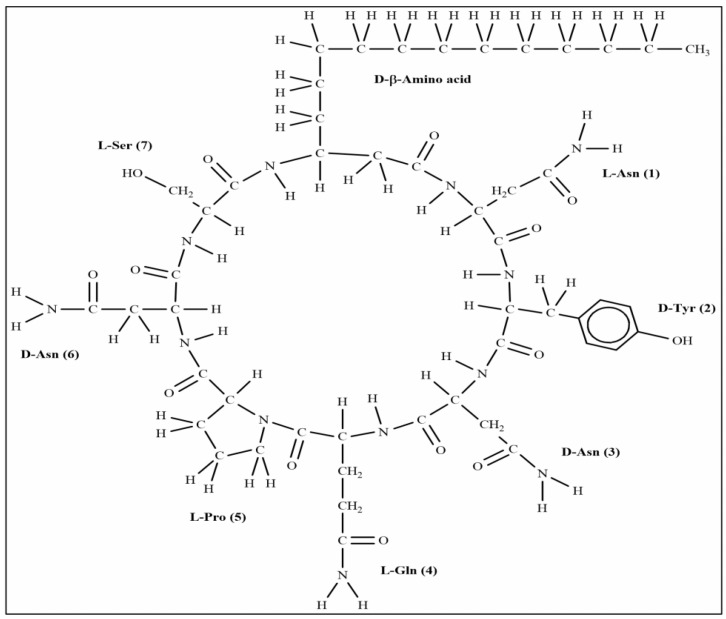
Structure of compound iturin A5 isolated from *B. amyloliquefaciens* S185.

The extracted compound **1** from S185 was shown as a white powder and directly identified using high-resolution electrospray ionization mass spectrometry (HR-ESI-MS): m/z [M + H]^+^, 1057.5671 (Figure 6). Based on this information, the molecular formula of this compound was predicted as C_49_H_76_N_12_O_14_ (Cacl. for C_49_H_77_N_12_O_14_^+^: 1057.5676). ^1^H nuclear magnetic resonance (NMR) reveals signals of the β-amino acid moiety (800 MHz, DMSO-d_6_, δ) (Figure S1): 7.11 (ovl, 1H, β-amino acid NH), 3.98 (m, 1H, β-amino acid C_3_H), 2.32 (d, 2H, β-amino acid C_2_H_2_), 1.41 (m, 2H, β-amino acid C_4_H_2_), 1.29–1.07 (m, β-amino acid aliphatic CH_2_), 0.83 (t, *J* = 6.4 Hz, 3H, β-amino acid C_15_H_3_). ^13^C NMR shows signals of (DMSO-d_6_, δ) (Figure S2): 45.8 (β-amino acid, C_3_), 42.2 (β-amino acid, C_2_), 35.0 (β-amino acid, C_4_), 31.8 (β-amino acid, C_13_), 29.6 (β-amino acid), 29.5 (β-aminoacid), 29.2 (β-amino acid), 29.1 (β-amino acid), 25.8 (β-amino acid, C_5_), 22.6 (β-amino acid, C_14_), 14.4 (β-amino acid, C_15_). All these data were compared with published NMR data and were consistent with the data for iturin A5 [33]. Thus, compound **1** was identified as iturin A5 (Figure 7, Table S4).

### 2.7. Iturin A5 Inhibited Spore Germination of Foc

**Figure 8:**
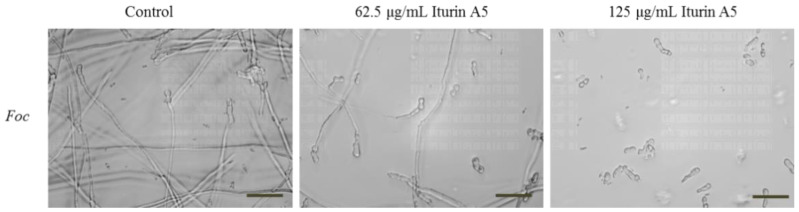
Inhibitory effect of antifungal compound iturin A5 on spore germination of *Fusarium oxysporum* f. sp. *cubense* (*Foc*). We incubated 2 × 10^5^ spores/mL of *Foc* with 0, 62.5 and 125 μg/mL of iturin A5 at 28 °C. After 24 h incubation, images were taken under an inverted microscope. Control is DMSO. Scale bar, 40 μm.

Despite the antifungal activity from iturin series is known, we assessed the effect of iturin A5 on the growth morphology of *Foc* under an inverted microscope. As shown in Figure 8, compared to the 100% germination rate of *Foc* in the absence of iturin A5, the germination rate of *Foc* decreased to 66% and 37% in the presence of 62.5 μg/mL and 125 μg/mL iturin A5, respectively (Table S5). Moreover, the germ tubes displayed significantly distorted morphology such as ballooned tips and loss of polarity (Figure 8).

## 3. Discussion

An essential goal of this study is focusing on the isolation and identification of the most effective biocontrol bacteria and antifungal compounds against *Foc*, the devastating pathogen for FWB. Marine microorganisms have been identified as promising natural sources for the development of biocontrol strains and agents [20]. In marine ecology, unusual circumstances such as extreme temperature and salinity provide a high success rate for the discovery of new and innovative microorganisms as well as their secondary metabolites. More than 20,000 distinct bioactive chemicals have been found in marine fauna and flora to date [34,35]. Previously, different species of *Bacillus* with confirmed biocontrol activity against *Foc* have been reported, including *B. amyloliquefaciens* GKT04 [36,37], W19 [38], NIN-6 [39], and NJN-6 [40], *B. velezensis* HN03 [41], and (iii) *B. subtilis* strains B26 [42], B04, B05 and B10 [43], and N11 [44]. However, an extensive screening of the biocontrol strains from marine bacteria to control FWB pathogen is limited.

*Bacillus* species are useful biocontrol candidates as they possess advantages of forming spores to survive for a long time in extreme environments, producing a wide range of physiologically active secondary metabolites that usually impede the growth of plant pathogens [45], and supporting the development and production of more stable commercial formulations over time from an agro-biotechnological perspective. Combining several *Bacillus* spp. strains is an attractive way to increase biocontrol efficiency under various cropping situations and environmental circumstances, given their adaptability and diverse biocontrol mechanisms. Because of the spore-forming abilities, the *Bacillus* group outperforms non-*Bacillus* species in terms of biocontrol efficiency. Spores can live in a variety of adverse conditions and remain stable during commercial production and maintain tolerance to fungicides [10,46]. *Bacillus*-mediated plant growth promotion is linked to the bacteria’s ability to produce phytohormones such as gibberellic acid and IAA, which improve host nutrient absorption and increase plant defense responses to biotic and abiotic stresses [47]. IAA produced by *B. amyloliquefaciens* FZB42 promotes root growth and development of lateral roots, resulting in increased nutrient intake from the rhizosphere [48]. Consistent with previous reports, in this study, we observed IAA production by strain S185 in the presence and absence of tryptophan in the medium. Several bacterial strains produce secondary metabolites such as HCN and ammonia, which are helpful in the management of fungal diseases in a variety of plants [31,49,50,51]. Similarly, the strain S185 showed strong ammonia production as well as potent antifungal activities against *Foc*, demonstrating the dual role of S185 in controlling FWB.

Being a potential biocontrol strain, one key essential is the colonization capacity of the strain to banana plants. Previously, CLSM of GFP-tagged strains was utilized to illustrate the colonization pattern of *B. megaterium* in rice [52], *B. megaterium* and *B. mycoides* in sugarcane [50]. Similarly, when using this strategy, we observed that the GFP-labelled S185 strain was successfully colonized in all banana plant tissues including leaves, stems, and roots (Figure 4). Previous studies have also suggested that strains with a wider metabolite tolerance are preferable for plant nodulation and growth [32,53]. The BIOLOG metabolic profiling study is a useful technique for identifying microbial diversity using different types of substrates. In this study, carbon utilization pattern of strain S185 was observed on the GNIII Biolog plate and the resulting phenotypes indicated that S185 can use a variety of carbon substrates (Table S2).

Variation in ambient temperatures between crop seasons influenced disease outcomes by affecting the biocontrol agent’s ability to kill pathogens [54]. In China, bananas are mostly grown in Guangdong, Guangxi, Hainan, Fujian, Yunnan, and Taiwan. However, diseases and adverse weather conditions continue to pose major obstacles to Chinese banana production. In this study, we found the strain S185 was able to grow between 20–50 °C, suggesting that the strain can operate consistently in temporally variable environments, thus making it a suitable biocontrol agent against *Foc* for naturally changing field circumstances. Wei et al. have reported that bacterial strains maintaining their activity in a variety of environments are promising candidates for consistent biocontrol applications [54].

Producing a wide range of secondary metabolites as active components is one of the main properties of *B. amyloliquefaciens*. Therefore, we performed the crude extract from S185 and examined antifungal activity against *Foc*. Not surprisingly, the extract displayed strong antifungal activity, suggesting antimicrobials in the S185 secondary metabolites. Subsequently, a compound **1** purified from the crude extract was identified using HR-ESI-MS (Figure 6). In combination with ^13^C- and ^1^H- NMR spectra the compound **1** was identified as iturin A5 (Figure 7).

Iturins are well-known antifungal metabolites generated by *Bacillus* strains [45,55,56], which comprise an amphiphilic peptide ring that is made up of seven chiral amino acids [57]. Iturin A, a member of the iturin family, has a high antibiotic action as well as a broad antifungal spectrum, making it a promising biological control agent for decreasing chemical pesticide use in agriculture [57]. Iturin A has also been found to be a useful drug in human and animal clinical studies because of its broad antifungal range, low toxicity, and low allergic impact [58]. Despite having so many benefits over chemical agents, iturin A has had very few practical uses too far, owing to poor strain productivity and very expensive manufacturing costs. To date, many iturin A producing strains have been identified such as *B. amyloliquefaciens*, *B. licheniformis*, *B. methyltrophicus*, *B. subtilis*, and *B. thuringiensis* [59,60,61,62,63]. However, this is the first report of iturin A5 lipopeptides produced from a thermotolerant marine *B. amyloliquefaciens* S185, which exhibited strong antifungal activity against *Foc*.

Iturin A disrupts the fungal cytoplasmic membrane by interacting mostly with ergosterol molecules, resulting in the formation of transmembrane channels that allow essential ions such as K^+^ to be released. For example, iturin A has been shown to change the permeability of membranes and the lipid content of *S. cerevisiae* cells [64]. Similarly, Hiradate et al. reported iturin A5 secreted by *B. amyloliquefaciens* RC-2 inhibited the development of mulberry anthracnose caused by the fungus, *Colletotrichum dematium* [33]. We also studied the antifungal activity of purified iturin A5 on the growth of *Foc* pathogen. Different concentrations of iturin A5 produced by S185 was given to test the growth of *Foc*, revealing that MIC of iturin A5 was 25 μg/disk for *Foc*. Earlier, MICs of iturin A for other pathogens were also measured, such as iturin A produced by *B. amyloliquefaciens* PPCB004 inhibited *F. aromaticum* and *Botryosphaeria* sp. with MICs of 1.0 and 1.5 mg/mL [65]. Aberrant conidial and spore germination in fungi was observed when treated by iturins produced by *Bacillus* strains [30,65,66]. Optical and fluorescent microscopy revealed morphological alterations in conidia and significant deformities in *F. graminearum* hyphae treated with iturin A [67]. Wang et al. observed *Phytophthora infestans* mycelia was damaged and had a rough and swollen shape after iturin A treatment [68]. Similarly to previous findings, we also observed the major changes and deformations of *Foc* hyphae after treatment with iturin-A5 produced by S185 (Figure 8).

## 4. Materials and Methods

### 4.1. Collection of Bacterial Strains

The sediment samples were collected from offshore of South Sea, China. The isolation procedure was described below. Ten grams of sediment from each sample were suspended in 90 mL of autoclaved seawater in a flask and shaken at 100 rpm for 1 h at 25 °C. Then, the mixture was serially diluted 10^−4^ times, and 100 µL of each dilution were distributed on 2216E agar plates. The colony morphology was evaluated by incubating all the plates at 25 °C for 2–4 days. Antagonistic activity was tested for all isolated strains against *Foc*. *B. amyloliquefaciens* S185 was chosen for identification. 16s *rRNA* sequence of S185 was submitted to the NCBI GenBank database under accession number MZ333473. All collected strains were stored in 25% glycerol solution at −80 °C.

### 4.2. In Vitro Screening for Antifungal, Plant Growth-Promoting (PGP) Traits, and Abiotic Stress Tolerance

A dual culture plate method was used to test the antifungal efficacy of strain S185 against *Foc*, following the procedure of Singh et al. [69]. Bacterial strain S185 was spotted 3 cm away from 5 mm diameter of actively growing fungal pathogens spotted in the center point of the yeast extract medium (YAG) (yeast extract-5 g, dextrose-20 g, 1000× trace element-1 mL, agar-18 g, and ultrapure water-1000 mL; 1000×trace element: ZnSO_4_·7H_2_O-2.2 g, H_3_BO_3_-1.1 g, MnCl_2_·4H_2_O-0.5 g, FeSO_4_·7H_2_O-0.5 g, CoCl_2_·5H_2_O-0.16 g, CuSO_4_-0.16 g, (NH_4_)_6_Mo_7_O_24_·4H_2_O-0.11 g, EDTA-5.0 g, and ultrapure water-100 mL). Plate containing only fungal disk served as the control and was incubated at 28 °C for 5–7 days. The percentage of inhibition was measured using the formula; [(R1−R2)/R1] × 100, where R1 is radial growth of the fungal pathogen in control plate and R2 is radial growth of fungal pathogen in the presence of test strain.

PGP traits including P-solubilization, production of IAA, siderophore, ammonia, and HCN by strain S185 were estimated by performing standard protocols of Brick et al. [70], Glickmann and Dessaux [71], Schwyn and Neilands [72], Dey et al. [73] and Lorck [74]. The ability of S185 to solubilize P was tested qualitatively using Pikovskaya medium added with tricalcium phosphate. Strain S185 was inoculated in the plate and observed for the clear hallow zone formation surrounding the isolate after 5–7 days incubation at 30 °C. IAA production was evaluated with colorimetric technique in the presence (0.5%) and absence of tryptophan in the medium. Siderophore production of strain S185 was detected on chrome azurol S (CAS) medium and the formation of a halo zone on the medium confirmed siderophore production. For ammonia production, strain S185 was cultured for 72 h in 10% sterile peptone water at 30 °C and changes in yellow color by adding 0.5 mL of Nessler’s reagent established the ammonia production. HCN production ability of strain S185 was tested on a nutrient broth (NB) medium containing 4.4 g/L glycine to produce hydrocyanic acid. A filter paper soaked with 0.5% picric acid and 2% Na_2_CO_3_ was placed on a cover plate, then sealed with Parafilm and incubated at 30 °C, and alteration of filter paper color displayed HCN production. All assays were repeated three times with five replications.

Strain S185 was further tested in NB for its capacity to survive under different abiotic stress conditions, such as pH, temperature, and NaCl, and the medium without inoculation was employed as a blank. Temperature tolerance was determined by incubating 0.1 mL fresh bacterial solution of strain S185 in NB medium (5 mL) for 36 h at 20, 25, 30, 35, 40, 45, and 50 °C in a shaker incubator at 120 rpm, and O.D. was measured at 600 nm. For pH tolerance, pH of the NB medium was adjusted to 1, 2, 3, 4, 5, 6, 7, 8, and 9 with sterile buffers. Fresh culture of strain S185 was placed into 5 mL of LB broth medium with varied pH levels and incubated at 37 °C in an incubator shaker at 120 rpm for 36 h, with growth monitored at 600 nm. In addition, the salt tolerance property of strain S185 was tested in 5 mL of NB medium supplemented with 1, 2, 3, 4, 5, 6, 7, 8, 9, 10, 11, and 12% of NaCl concentration. 0.1 mL bacterial suspension (0.1 mL) was transferred in NB tubes and kept at 37 °C at 120 rpm in a shaker incubator and growth was measured after 36 h at 600 nm.

### 4.3. Phylogenetic Study of Strain S185

Phylogenetic analysis of strain S185 based on 16S *rRNA* sequences was performed with NCBI GenBank reference sequences. Sequence alignment was performed using the ClustalW and BlastN search algorithms, and closely related sequences were retrieved from NCBI. The Neighbor-Joining approach [75] was used to prepare phylogenetic analysis based on 16S *rRNA* sequence using MEGAX version X [76]. The number of differences method [77] was used to compute the genetic evolutionary distances, and the bootstrap test (1000 replicates) was performed as stated by Felsenstein [78].

### 4.4. Carbon Utilization Pattern of Strain S185

Carbon substrates utilization pattern of strain S185 was studied using GENIII BIOLOG^(R)^ Phenotype Micro-ArrayTM plate. Strain S185 was cultivated at 30 °C on Luria-Bertani (LB) agar medium, then washed and suspended in an inoculation fluid (IF) to achieve a transmittance of 90–98 percent as per the protocol. The phenotypic fingerprint was formed by incubating a 100 µL of cell suspension in the 96 wells of the GNIII Micro-Plate at 30 °C for 48 h. During incubation, the wells’ respiration rate increases, allowing the cells to use a variety of carbon sources while growing. Increased respiration causes the tetrazolium dye to be reduced, resulting in purple color. Readings were recorded using an automated BIOLOG(R) Micro-Station Reader following the manufacturer guidelines after incubation.

### 4.5. Tagging of S185 with GFP-pPROBE-pTetr-TT

The pPROBE-pTetr -TT plasmid expressing green fluorescent protein (GFP) was provided by the Agriculture College, Guangxi University, Nanning, China. Tissue culture banana plantlets obtained from Guangxi Academy of Agricultural Sciences, Nanning, China were used for this experiment. Freshly grown cultures of S185 and GFP-pPROBE-pTetr-TT in LB medium were mixed (1:2) and then incubated for 36–48 h at 32 °C with a 160 rpm orbital shaker. Following the incubation, 100 µL of bacterial broth was disseminated on LB agar plate and kept overnight to assess the purity of the tagged strain, as well as validate the tagging using confocal laser scanning microscopy (CLSM). Fluorescent strain S185, when exposed to UV light, was chosen for further investigation.

#### Colonization of S185 in Banana Plantlets

Before bacterial inoculation, banana tissue culture plantlets were rinsed with autoclaved distilled water. Plantlets were placed in an autoclaved glass container comprising 50 mL of MS liquid media (mixture of basal salt and sucrose) at 30 °C in a growth chamber. After 3–4 days, plantlets were carefully moved to a new bottle comprising tagged bacterial suspension (∼2.0 × 10^5^/mL cell count). The uninoculated banana plantlets (control) were placed in a growth chamber set at 30 °C with a 14 h photoperiod and 60 µmol·m^−2^·s^−1^ photon flux density. Plantlets were removed after 72–96 h of growth, rinsed with distilled water, and CLSM was used to check for bacterial colonization. Banana leaf, stem, and root (inoculated and uninoculated) samples were sliced into 1 cm lengths and put on the bridge slide with 10% (*v*/*v*) of glycerol before being detected with CLSM at varying emission rates depending on the intensity of autofluorescence UV light (Leica DMI 6000, Mannheim, Germany) [79].

### 4.6. Cultivation and Extraction of Antifungal Compounds

Strain S185 was cultured in 12 L of YAG medium at 25 °C for 9 days. The solid fermentation products were cut into small pieces and carefully extracted with ethyl acetate (EtOAc)/methanol (MeOH)/acetic acid (HAc) solution (80:15:5, *v*/*v*/*v*). The extraction was performed three times for the preparation of crude extracts. Crude extracts were dissolved in water and first extracted three times with EtOAc and then extracted three times with n-butanol. We obtained 7.981 g of EtOAc extract and 4.674 g of n-butanol extract after condensation and evaporation with a rotary evaporator. The n-butanol extract residue (4.674 g) was purified by Sephadex LH-20 (Amersham Pharmacia) (MeOH, 2 × 100 cm, 1 mL·min^−1^) to produce 6 fractions (Fr.1.1-Fr.1.6). Fr.1.2 (592.7 mg) was applied to a silica gel column (Qingdao Marine Chemical Factory) (GF254, 3 × 50 cm, 70 g) using a chloroform: methanol (90:10, 80:20, 70:30) solvent gradient system with ammonia water (0.3%) to generate fractions Fr.1.2.1-Fr.1.2.5. Finally, Fr.1.2.5 (326 mg) was purified by Sephadex LH-20 (MeOH, 2 × 100 cm, 1 mL·min^−1^) to obtain compound **1** (240.8 mg).

#### 4.6.1. Antifungal Assay of Extracted Compound **1**

Antifungal activity of selected compound **1** was assessed by disk diffusion method against pathogenic fungi *Foc* [80]. The extracted compound was dissolved in methanol and added in 6 mm diameter of paper disks at different levels of concentrations, i.e., 25, 50, 100, 200, and 400 μg/disk, respectively. A disc containing only methanol was used as the control. The dried paper discs were applied onto the surface of YAG plates dispersed with *Foc* at 28 °C for 24 h to 48 h. After incubation, the diameter of the inhibition zone was determined. Each treatment was repeated three times.

#### 4.6.2. Structure Identification of Compound **1**

The Nuclear Magnetic Resonance (NMR) spectra were generated using an Agilent NMR system 800 MHz NMR spectrometer (Agilent Technologies Inc., Colorado Springs, CO, USA) to determine the mass of the antifungal compound **1**. Electrospray ionization mass spectrometry (ESI-MS) and high-resolution electrospray ionization mass spectrometry (HR-ESI-MS) were performed using a Thermo Scientific Orbitrap Elite MS spectrometer (Thermo Fisher Scientific, Waltham, MA, USA) to identify the structure of compound **1** isolated from S185.

### 4.7. Effect of Purified Iturin A5 on Spore Germination of Foc

The inhibitory effect of purified iturin A5 on spore germination of *Foc* was performed following the standard EUCAST MIC determination method [81] and observed under an inverted microscope (Leica Microsystems CMS GmbH, Ernst-Leitz-Str. 17–37, D-35578, Wetzlar). *Foc* was cultured on a YAG medium at 28 °C for 7 days and then mixed with 10 mL of 0.2% Tween-80 solution. Subsequently, we gently scraped the surface of the fungal colony with a sterile loop, and spores were collected. Finally, the spore concentration was diluted to 2 × 10^5^ CFU/mL. 10 mg iturin A5 was dissolved in 1 mL DMSO to obtain the concentration of 10 μg/μL stock solution. 2 × RPMI1640 medium with 2% glucose was used for spore germination, which was carried out in 96-well plates. Firstly, 300 μL liquid spores were added to the Eppendorf tube and mixed with 270 μL of 2 × RPMI1640 medium with 2% glucose. For control, 30 μL DMSO was added to the Eppendorf tube. For treatment, different concentration of iturin A5 (62.5, 125, 250, and 500 μg/mL) was used. 200 µL of prepared different spore suspensions were added into 96-well plates. After 24 h of incubation at 28 °C, spore germination was observed under an inverted microscope.

## 5. Conclusions

*Bacillus* strains are potential candidates for controlling FWB because of their wide range of secondary metabolites to inhibit the growth of pathogens as well as colonization and PGP properties. Searching for cost-effective and environmentally friendly *Bacillus* to manage banana pathogenic outbreaks is urgent and practical. In this study, a marine bacterium, *B. amyloliquefaciens* S185, exhibited thermotolerance, IAA, and ammonia production, diverse carbon utilization patterns, and good colonizing capacity in banana plants. In parallel, the S185 strain displayed strong antagonistic activity against *Foc*. The compound iturin A5 from S185 is the main bioactive component for antifungal activity by delaying spore germination and disrupting the polarity establishment of *Foc*. In summary, strain S185 and its iturin A5 lipopeptide is a promising biocontrol strain and agent for FWB. However, field testing of this strain is required to evaluate its efficacy in controlling Foc and promoting banana growth in the future.

## Data Availability

Data sharing does not apply to this article as no new data were created or analyzed in this study.

## Supplementary Materials

The following are available online at https://www.mdpi.com/article/10.3390/md19090516/s1, Figure S1: The ^1^H spectra of compound **1** in DMSO-d_6_. Figure S2: The ^13^C spectra of compound **1** in DMSO-d_6_. Table S1: Antifungal and plant growth promoting features of thermotolerant marine *Bacillus amyloliquefaciens* S185. Table S2: List of carbon sources utilized by S185 in GNIII Biolog plate. Table S3: Inhibitory effect of compound **1** extracted from S185 on the growth of *Fusarium oxysporum* f. sp. *cubense*. Table S4: NMR spectroscopic data for compound **1**, iturin A2 and iturin A5 in DMSO-d_6_. Table S5: Effect of iturin A5 on spore germination rate of *Fusarium oxysporum* f. sp. *cubense*.
