# Supplementary material for: A Thermotolerant Marine Bacillus amyloliquefaciens S185 Producing Iturin A5 for Antifungal Activity against Fusarium oxysporum f. sp. cubense
Source: Mar Drugs. 2021 Sep 11;19(9):516. doi: 10.3390/md19090516 (PMC8472358; doi:10.3390/md19090516)
Supplement: Supplementary file 1 [file marinedrugs-19-00516-s001.zip › marinedrugs-1364165-supplementary.pdf]

Figure S1. The ^{1}H spectra of compound 1 in DMSO-d6.

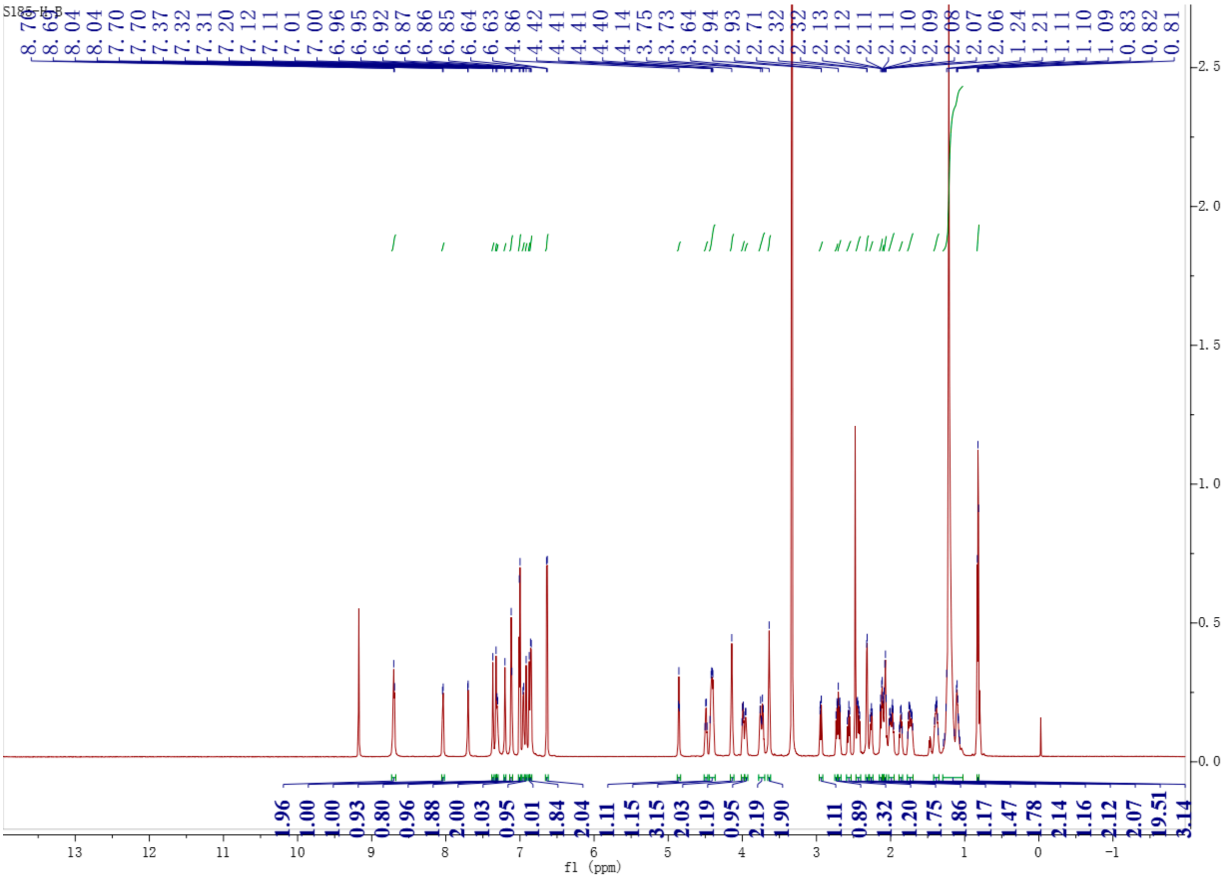

Figure S2. The ^{13}C spectra of compound 1 in DMSO-d$_6$.

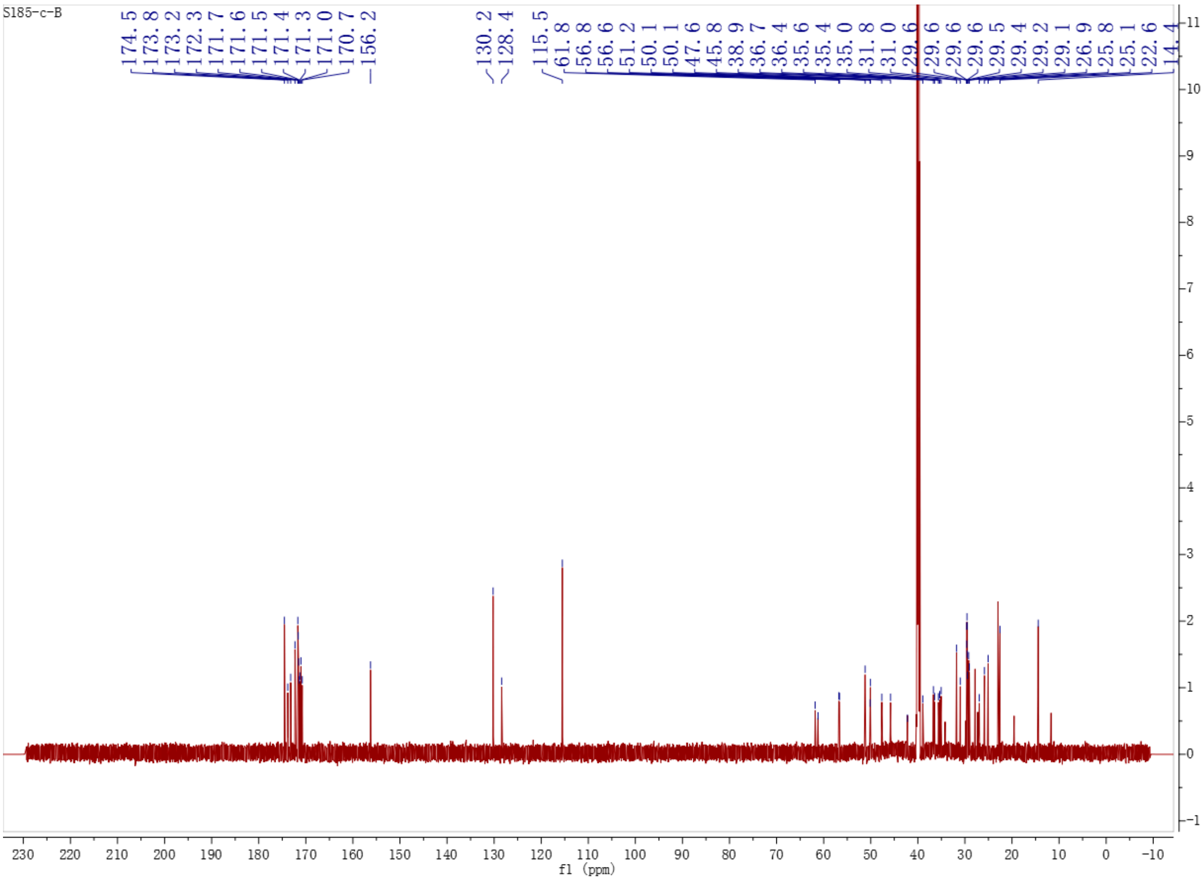

Table S1. Antifungal and plant growth promoting features of thermotolerant marine *Bacillus amyloliquefacien*s S185.

**Parameters**	***Bacillus amyloliquefacien*s S185**
**Antifungal Activity (% Inhibition)**	
Fusarium oxysporum f. sp. *cubense*	78
**Plant Growth Promoting Traits**	
Siderophore production	-
Phosphate solubilization	-
HCN production	-
Ammonia production	+
**Indole Acetic Acid (μg ml^{-1})**	
Absence of Tryptophan	57.13 ± 1.32
Presence of Tryptophan (0.5 %)	93.96 ± 2.28

(+) = Positive; (-) =Negative

Table S2. List of carbon sources utilized by S185 in GNIII Biolog plate.

S. No.	Carbon Sources
1	Dextrin
2	D-Turanose
3	D-Salicin
4	N-Acetyl-D-Glucosamine
5	N-Acetyl-β-DMannosamine
6	N-Acetyl-D-Galactosamine
7	N-AcetylNeuraminic Acid
8	D-Fructose
9	D-Galactose
10	3-Methyl Glucose
11	D-Fucose
12	L-Fucose
13	Inosine
14	D-Sorbitol
15	D-Mannitol
16	D-Arabitol
17	myo-Inositol
18	Glycerol
19	D-Glucose-6-PO_4
20	D-Fructose-6-PO_4
21	D-Aspartic Acid
22	D-Serine
23	Glycyl-L-Proline
24	L-Alanine
25	L-Arginine
26	L-Aspartic Acid
27	L-Glutamic Acid
28	L-Histidine
29	L-Serine
30	Lincomycin
31	Niaproof 4
32	Pectin
33	D-Gluconic Acid
34	D-Glucuronic Acid
35	Glucuronamide
36	Mucic Acid
37	Quinic Acid
38	Tetrazolium Violet
39	Tetrazolium Blue
40	L-Lactic Acid

Table S3. Inhibitory effect of compound 1 extracted from S185 on the growth of *Fusarium oxysporum* f. sp. *cubense.*

**Concentration of compound 1 (μg/disk)**	**Diameter of inhibition zone(mm)**
0 (Control)	-
25	7.67
50	9.67
100	12.33
200	13.33
400	14.33

(-)=Negative

Table S4. NMR spectroscopic data for compound 1, iturin A2 and iturin A5 in DMSO-d_6.

Position	Compound 1		Iturin A2		Iturin A5	
	δ_C	δ_H, m	δ_C	δ_H, m	δ_C	δ_H, m
1	51.2	4.44, m	50.8	4.42, m	50.8	4.42, m
2	36.7	2.28, dd	36.3	2.29, dd	36.3	2.29, dd
		2.14, m		2.16,dd		2.16,dd
3	171.3		170.8		170.8	
4	173.8		173.3		173.3	
1-NH		7.70, d		7.70, d		7.70, d
3-NH_2		7.32, s		7.31, s		7.31, s
		6.92, s		6.89, s		6.89, s
5	56.8	4.00, m	56.3	4.02, m	56.3	4.02, m
6	35.4	2.95, dd	34.9	2.96, dd	34.9	2.96, dd
		2.74, m		2.73, dd		2.73, dd
7	128.4		127.9		127.9	
8	130.2	7.01, d	129.7	7.01, d	129.7	7.01, d
9	115.5	6.64, d	115.0	6.65, d	115.0	6.65, d
10	156.2		155.8		155.8	
11	115.5	6.64, d	115.0	6.65, d	115.0	6.65, d
12	130.2	7.01, d	129.7	7.01, d	129.7	7.01, d
13	171.6		171.1		171.1	
5-NH		8.70, d		8.68, d		8.68, d
14	51.2	4.44, m	50.8	4.43, m	50.8	4.43, m
15	36.4	2.58, dd	36.0	2.58, dd	36.0	2.58, dd
		2.46, m		2.47, dd		2.47, dd
16	171.7		171.2		171.2	
17	170.7		170.3		170.3	
14-NH		8.04, d		8.04, d		8.04, d
16-NH_2		7.20, s		7.19, s		7.19, s
		6.87, s		6.85, s		6.85, s
18	50.1	4.50, m	49.6	4.51, m	49.6	4.51, m
19	26.9	2.02, m	26.5	2.03, m	26.5	2.03, m
		1.77, m		1.75, m		1.75, m
20	31.0	2.09, m	30.5	2.09, m	30.5	2.09, m
21	174.5		174.0		174.0	
22	171.5		171.0		171.0	
18-NH		6.96, d		6.91, d		6.91, d
21-NH_2		7.12, s		7.10, s		7.10, s
		6.86, d		6.82, s		6.82, s
23	61.2	4.14, s	60.8	4.16, m	60.8	4.16, m
24	29.4	2.11, m	29.0	2.12, m	29.0	2.12, m
		1.77, m		1.74, m		1.74, m
25	25.1	1.98, m	24.6	1.98, m	24.6	1.98, m
		1.88, m		1.88, m		1.88, m
26	47.6	3.76, m	47.2	3.75, m	47.2	3.75, m
27	173.2		172.7		172.7	
28	50.1	4.44, m	49.7	4.42, m	49.7	4.42, m
29	35.6	2.71, dd	35.2	2.71, dd	35.2	2.71, dd
		2.46, m		2.45, dd		2.45, dd
30	172.3		171.8		171.8	
31	171.4		170.9		170.9	
28-NH		8.70, d		8.68, d		8.68, d
30-NH_2		7.37, s		7.35, s		7.35, s
		6.86, d		6.82, s		6.82, s
32	56.6	4.14, s	56.1	4.16, m	56.1	4.16, m

33	61.8	3.64, m	61.3	3.66, d	61.3	3.66, d
34	171.0		170.6		170.6	
32-NH		7.31, d		7.31, d		7.31, d
33-OH		4.86, m		4.83, br		4.83, br
35	45.8	3.98, m	45.3	3.97, m	45.4	3.97, m
36	42.2	2.32, d	41.7	2.33, m	41.7	2.33, m
37	171.7		171.2		171.2	
38	35.0	1.41, m	34.6	1.40, m	34.6	1.40, m
39	25.8	1.29-1.07, m	25.3	1.28-1.08, m	25.3	1.28-1.08, m
40-45	29.1-29.6	1.29-1.07, m	28.6-29.1	1.28-1.08, m	28.6-29.1	1.28-1.08, m
46	29.1-29.6	1.29-1.07, m	31.3	1.28-1.08, m	28.6-29.1	1.28-1.08, m
47	31.8	1.29-1.07, m	22.0	1.28-1.08, m	31.2	1.28-1.08, m
48	22.6	1.29-1.07, m	13.9	0.84, t	22.0	1.28-1.08, m
35-NH		7.11, ovl		7.12, d		7.12, d
49	14.4	0.83, t			13.9	0.84, t

Note: Only the complete NMR data of iturin A2 were reported [33]. Iturin A5 shares the same skeleton structure as iturin A2 shown by the red labeled δ_C and δ_H signals, the different β-amino acid moiety of iturin A5 was shown by the green labeled δ_C and δ_H signals. NMR spectroscopic data of compound 1 were corresponding to the signals of iturin A5, therefore compound 1 was identified as iturin A5. Ovl: overlapped with other signals.

Table S5. Effect of iturin A5 on spore germination rate of *Fusarium oxysporum* f. sp. *cubense*

Concentration of iturin A5 (μg/ml)	Spore germination rate (%)
0 (Control)	100
62.5	66
125	37

Iturin A5 was dissolved in DMSO. Both control and treatment groups contained 5% DMSO.
